# Coupling between Ion Drift and Kinetics of Electronic Current Transients in MAPbBr_3_ Single Crystals

**DOI:** 10.1021/acsenergylett.1c02578

**Published:** 2022-02-11

**Authors:** Marisé García-Batlle, Javier Mayén Guillén, Marian Chapran, Oriane Baussens, Julien Zaccaro, Jean-Marie Verilhac, Eric Gros-Daillon, Antonio Guerrero, Osbel Almora, Germà Garcia-Belmonte

**Affiliations:** † Institute of Advanced Materials (INAM), Universitat Jaume I, 12006 Castelló, Spain; ‡ Grenoble Alpes University, CEA, LITEN, DTNM, F38000 Grenoble, France; § Grenoble Alpes University, CEA, LETI, DOPT, F38000 Grenoble, France; ∥ Grenoble Alpes University, CNRS, Grenoble INP, Institut Néel, F38042 Grenoble, France

## Abstract

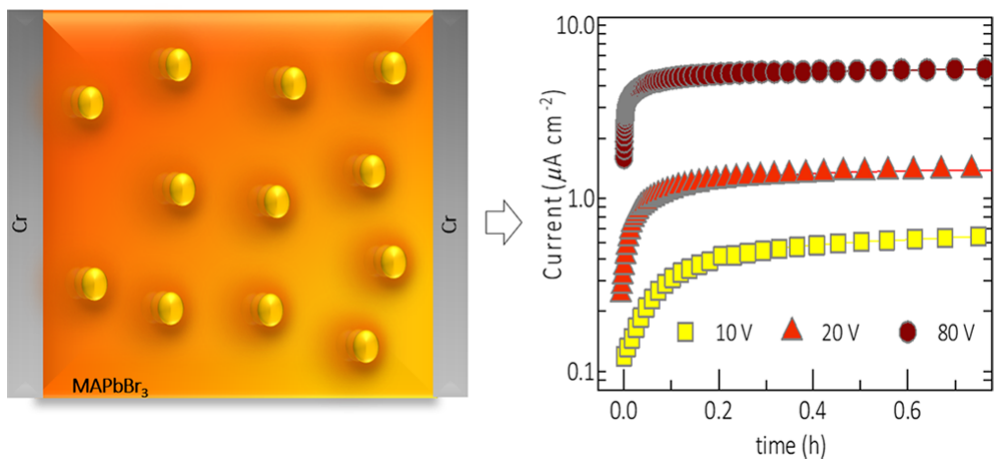

The optoelectronic properties of halide perovskite materials have fostered their utilization in many applications. Unravelling their working mechanisms remains challenging because of their mixed ionic–electronic conductive nature. By registering, with high reproducibility, the long-time current transients of a set of single-crystal methylammonium lead tribromide samples, the ion migration process was proved. Sample biasing experiments (ionic drift), with characteristic times exhibiting voltage dependence as ∝ *V*^–3/2^, is interpreted with an ionic migration model obeying a ballistic-like voltage-dependent mobility (BVM) regime of space-charge-limited current. Ionic kinetics effectively modify the long-time electronic current, while the steady-state electronic currents’ behavior is nearly ohmic. Using the ionic dynamic doping model (IDD) for the recovering current at zero bias (ion diffusion), the ionic mobility is estimated to be ∼10^–6^ cm^2^ V^–1^ s^–1^. Our findings suggest that ionic currents are negligible in comparison to the electronic currents; however, they influence them via changes in the charge density profile.

The outstanding and versatile optoelectronic properties of halide perovskite thin films, most typically based on methylammonium lead triiodide, have allowed their utilization in many applications, such as solar cells, light-emitting diodes, photodetectors, and lasers.^1,2^ In the case of halide perovskite single crystals (SCs), the promising sensitivity and favorable characteristics (high absorption coefficient, long carrier diffusion length, and long carrier lifetime) have motivated arduous research in the field of high-energy radiation detectors.^3−5^ Particularly, the use of methylammonium lead tribromide (MAPbBr_3_) SCs for radiation detectors has recently shown significant progress^6−8^ because of their easy solution-based fabrication methods and the resulting high crystalline quality and material stability.^9−11^ Nevertheless, unravelling the working mechanisms of halide perovskite-based devices remains challenging because of their mixed ionic–electronic conductive nature, which usually complicates the comprehension of certain response features.^12,13^ Moreover, understanding ion-originated modulations of the electronic properties is essential to further progress into the physics and operating modes of halide perovskite devices.^14−16^

The ion migration in MAPbBr_3_ SCs has been investigated through different methods (see Table S1 in the Supporting Information).^17−20^ Several hypotheses have been suggested to distinguish ionic from electronic contributions to the measured current density (*J*) flowing through perovskite-based samples.^20−25^ Nevertheless, there is no conclusive evidence nor consensus on the most appropriate model. The major issues are the overlapping time scales of both ionic and electronic phenomena, giving rise to the so-called hysteresis of halide perovskites,^26^ and the materials reactivity causing performance degradation. Both phenomena hinder the interpretation of many experiments because of signal instability, lack of reproducibility, and strong dependency on several parameters, such as the applied voltage (*V*), polarization history, temperature (*T*),^27^ and moisture. Accordingly, for any study on the electrical response of mixed ionic–electronic halide perovskite it is essential to (i) identify a state (or regime) where one of the two contributions can be negligible, (ii) validate reproducibility, and (iii) check whether material or interface degradation may affect the conclusions.

In order to explore purely electronic currents, one can either analyze a signal so fast that the ions cannot follow or so slow that a steady-state is attained and the ionic displacement currents no longer contribute to the total direct-current-mode (DC) flux of charge carriers. High-frequency perturbation studies are an example of fast signal where the mobile ions are kept “frozen” in a homogeneous quasi-equilibrium state. As such, these studies are useful for evaluating the electronic phenomena without ionic contributions to the current or the charge density profile. However, they do not correspond to a realistic situation for device operation. In practical applications, the total DC currents are affected by the coupled contributions of electrons and ions to the charge density profile, hence defining the magnitude and time evolution of the total current.

In this work, an experiment is presented that consists of registering the long-time current transient response to different biases for a set of MAPbBr_3_ SC samples, symmetrically contacted with chromium electrodes. The measured current exhibits exponential rise until saturation at the steady state. On the other hand, relaxation under zero bias for enough time allows reaching equilibrium, which ensures negligible electrostatic potential energy before subsequent biasing. Thereby, a specific biasing protocol is followed that guarantees sufficient stability and permits high reproducibility. Ion migration is proved by either electrical field drift (current transient experiments) or ion diffusion (zero-bias impedance spectroscopy). As expected, dissimilar time scales are encountered for ion movement because of a change in transporting driving force. Experiments are analyzed either by means of the ionic dynamic doping model (IDD)^17,21^ or the model of ballistic-like voltage-dependent mobility (BVM)^28^ regime of space-charge-limited currents (SCLCs) accounting for the separate regimes of ion diffusion and ion drift, respectively. Our findings suggest an ionic–electronic coupling in which purely electronic currents are measured that follow the slow kinetics of mobile ion redistribution. Ionic mobility values in the order of μ_*i*_ ≈ 10^–6^ cm^–2^ V^–1^ s^–1^ result in a consistent way through our analysis.

## Experimental Methods

Single-crystals of MAPbBr_3_ were prepared following the inverse temperature crystallization (ITC) growth method.^10^ The precursors MABr and PbBr_2_ were dissolved in *N*,*N*-dimethylformamide (DMF) with an equimolar ratio. The precursor solution containing a seed crystal was placed in an oil bath whose temperature was programmatically raised from room temperature to 85 °C. Finally, the obtained high-quality MAPbBr_3_ single crystals were polished and then contacted with chromium electrodes. This metal has been selected because it spontaneously oxidizes during sample preparation giving rise to a thin layer of Cr_2_O_3_,^29^ preserving the contact from rapid chemical degradation. The thicknesses *L* of the samples ranged from 0.96 to 2.20 mm. The evaporated electrodes had an active area *A* of ∼20 mm^2^.

The single crystals were characterized by optical transmission spectroscopy (Figure S1) and X-ray powder diffraction (Figure S2). The transmission spectrum was recorded on a Perkins-Elmer Lambda 900 spectrometer, using an unpolarized beam. Polished samples were mounted in front of an aperture with a diameter of 2 mm. The optical band gap, extracted from Tauc plot, shows a value of 2.21 eV. Structural characterizations were made using a D8 Endeavor diffractometer equipped with a Johanssonn monochromator. The single-crystal sample was grinded into powder and measured in the Bragg–Brentano θ–2θ geometry. The diffractogram reveals the standard cubic space group *Pm*3*m* of MAPbBr_3_ crystals with lattice dimension *a* = 5.928 Å, and without any trace of secondary phase.

Chronoamperometry experiments were carried out with a Source Measure Unit Keithley Model 2612B, and impedance spectroscopy (IS) measurements were conducted with a PGSTAT302N potentiostat from Metrohm AUTOLAB. Current measurements were carried out under several long-time direct-current mode (DC) bias conditions in the ranges of ±200 to 0 V. The samples were kept in the dark at 300 K with N_2_ circulation for preventing humidity- and oxygen-induced degradations.

## Measurement of Current Transients

The *J*–*V* characteristics of MAPbBr_3_ SC samples are shown in Figure S3. One can infer from the linearity of the *J*–*V* response an ohmic behavior in the selected voltage range, without significant evidence of hysteretic current contribution. In fact, at the used scan rate of 90 mV/s, the electronic current is instantly observed because of a much faster speed of electrons/holes compared with that of ions.^30^ However, it is not evident that the *J*–*V* responses have necessarily reached the steady-state regime, which might require much longer polarization times.

**Figure 1:**
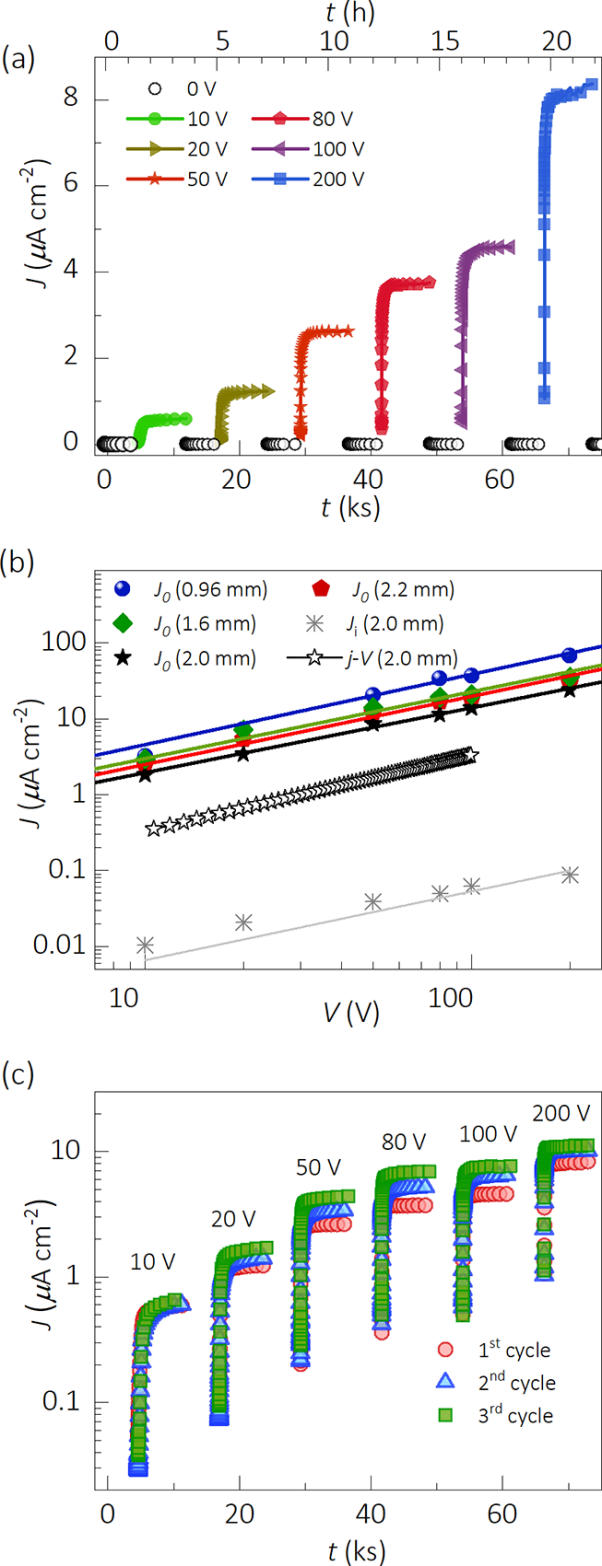
Long-time current evolution upon a biasing protocol for a ∼2 mm-thick MAPbBr_3_ SC sample. (a) Full biasing routine of one cycle. After each bias, the device is kept under short-circuit (0 V bias) conditions to observe the relaxation current (Figure S4). (b) Variation of the saturation current of four SC samples of different thicknesses is shown with the corresponding linear fitting (solid lines); one of the *J*–*V* curves is registered at high scan rates, and the ionic drift currents *J*_i_ are immediately obtained after bias removal (Figure S3). (c) Biasing responses of three consecutive cycles. Replicas of this experiment with other samples are shown in Figure S7.

Figure 1a displays the current response of a MAPbBr_3_ SC sample upon the application (solid dots) and removal (open dots) of step voltages (10, 20, 50, 80, 100, and 200 V). In this biasing protocol, after each 9600 s interval of positive bias, the crystal relaxes at 0 V for 3000 s. At this zero-bias, a small current undershoot appears of the order of nA cm^–2^ with the opposite sign and long decay time (see Figure S4). As expected, that negative current seems to exclusively obey the ion dynamics.^30^

In the zero bias, recovering experiment, the IS spectra were measured as a function of time *t* after a DC polarization of 10 V and fitted to the equivalent circuit of Figure S5. The high-frequency resistance (*R*) values were normalized and shown to follow a trend *R* ∝ *t*^*1/2*^ (see Figure S6), in agreement with the IDD model (see section S2 of the Supporting Information).^17,21^

It is of major importance to study the drift current upon biasing. In Figure 1a, the electronic current density (*J*_e_) exponentially rises and finally saturates approaching steady-state values *J*_0_ at long times (10–1000 s). We interpret the current level as being originated by electronic (electrons and holes) carriers. However, such long response times suggest that the kinetics of current saturation is governed by the slow movement of ionic species, then entailing a coupling between electronic drift and ionic transport. The *J*_0_–*V* curve is shown in Figure 1b for four samples of different thicknesses along with a *J*–*V* curve (Figure S3) for comparison. From the allometric fitting, a *J*_0_ ∝ *V*^*n*^ law is extracted with the power *n* = 0.96 ± 0.07, which is more likely due to the occurrence of an ohmic conduction regime for the electronic carriers. It is also worth noting that fast *J*–*V* curves exhibit lower current values than those extracted from the steady-state regime.

As shown in Figure 1c, the measurement procedure from 10 to 200 V bias was repeated three consecutive times. Note that the steady-state current slightly increases between cycles while keeping the biasing protocol. Replicas of this experiment with other samples of similar thickness are shown in Figure S7.

**Figure 2:**
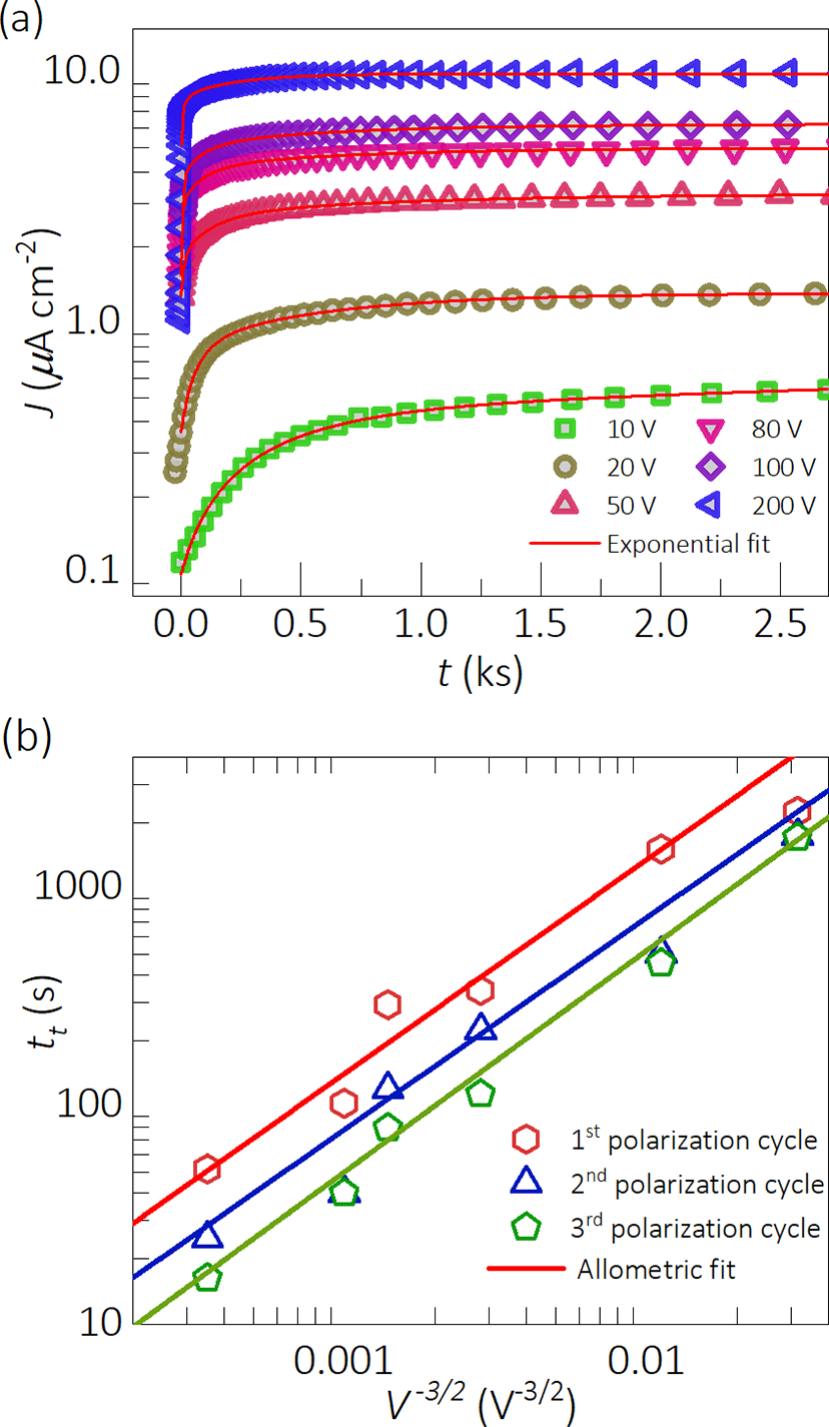
Parameterization of the long-time current transient response to different voltage steps during the first cycle of measurement of a ∼2 mm-thick MAPbBr_3_ SC sample: (a) experimental current transient (dots) and exponential fittings (lines) and (b) corresponding characteristic ionic relaxation time constants (dots) and allometric fittings (lines) as a function of the applied voltage.

To quantify the ionic kinetics and estimate ionic transport parameters under different biases, we explored the shape and magnitude of the current transients. Figure 2a shows the current response (dots) with the corresponding exponential fittings (lines) for the first cycle (see fitting results for the other two cycles in Figure S8). The exponential rise can be explicitly expressed as *J*_e_(*t*) *= J*_0_(1 – exp[−*t*/τ]) where τ indicates the time constant of the ion migration process. Because τ accounts for only 63% of the variation, we rather use the total characteristic time *t*_t_*=* 4τ, which corresponds to 98% of the transition to the steady-state. Then, by plotting the resulting time constants as a function of the applied bias at the power of −3/2, a clear *t*_t_ ∝ *V*^–3/2^ relationship is identified in Figure 2b. Importantly, also illustrated in Figure 2b, this behavior is even observed after 3 consecutive cycles of measurement, indicating a significant reproducibility.

## Analysis of Ionic Transit Time

The long-time current transient experiments result in two main trends: *J*_0_ ∝ *V*^*n*^, with *n* ∼ 1 and *t*_t_ ∝ *V*^–3/2^. The ohmic behavior of the saturated current as a function of voltage indicates that, once the steady-state is attained, there is no significant influence of the actual charge density profile on the electronic currents. Importantly, that ohmic regime is obeyed irrespective of the sample thickness (Figure 1b). Hence, *J*_0_ variation on biasing cycle (Figure 1c) is caused by either (i) an interface phenomenon which merely modified the contact resistance and/or (ii) a bulk issue directly associated with the homogeneous change of defect density.

Ionic mobility can be directly evaluated by analyzing the resistance response under the diffusion-controlled relaxation at *V* = 0 V. By using the IDD model (see Section S2),^17,21^ the evolution of *R* with time from the IS characterization results in an ionic diffusion coefficient as *D*_i_ = (3.1 ± 0.4) × 10^–8^ cm^2^ s^–1^, which corresponds to ion mobilities of about μ_i_ = (1.20 ± 0.15) × 10^–6^ cm^2^ V^–1^ s^–1^ by using Einstein’s relation (eq S8). Moreover, the crystal electronic conductivity can be inferred from the linear relationship *R* ∝ *L*/*A* (see Figure S9) and results in σ_e_ = (20 ± 5) × 10^–8^ Ω^–1^ cm^–1^, also in agreement with previous reports.^21^ In addition, high electronic mobility (μ_e_) has been measured using laser time-of-flight techniques in our samples being μ_e_ = 17 cm^2^ V^–1^ s^–1^ (see Figure S10) with a similar procedure as the one previously reported.^19^ Note therefore the huge difference in mobility between ionic species and electronic carriers. In fact, it makes sense to estimate the order of magnitude of the ionic drift currents from the maximum diffusion currents in each short-circuit period of the experiments (from Figure S4). The comparison is made in Figure 1b, which indicates that *J* is greater than *J*_i_ by more than 2 orders of magnitude. This would indicate that ionic currents, although present, should be negligible as part of the total current measured during the relaxation toward the steady state (i.e., *J*_i_ ≪ *J*), which have to be considered as being mainly dominated by electronic carriers (*J* ≃ *J*_e_). The difference in current level should also apply for the respective conductivity.^13^ However, the measured time constants within the range of 10–1000 s (Figure 2b) readily suggest that the transition toward the steady state carries the information on the ionic kinetics.

**Figure 3:**
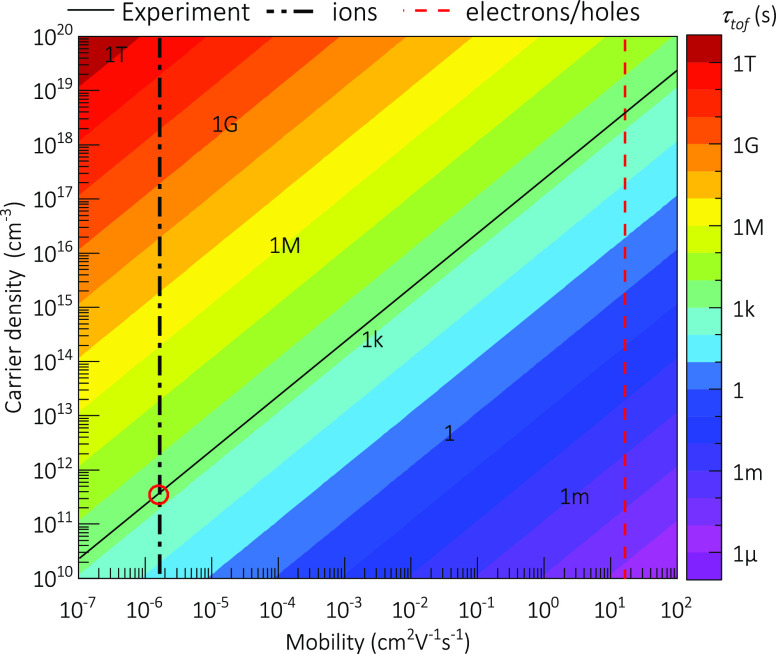
Time of flight (right-hand color bar) as a function of mobility and effective charge carrier density for the BVM regime of SCLC as in eq 1. Parameters as for MAPbBr3 single crystal: *V* = 100 V, *L* = 2 mm, ϵ_r_ = 76,^21^*V*_0_ = 10 V. Note that the charge carrier density axis may refer to either ionic or electronic charge carriers in each case.

The behavior of the ionic-related relaxation times *t*_t_ ∝ *V*^–3/2^ resembles that of the τ_tof_ in the classic Child–Langmuir law^31,32^ for the ballistic SCLC (see section S3), which suggests the occurrence of ionic currents with a *J*_i_ ∝ *V*^3/2^ trend. The ballistic SCLC tackles the space charge modification when the electrostatic energy is totally converted into kinetic energy upon application of an external electric field. Consequently, the drift velocity relates with the electrostatic potential as *v*_d_ ∝ φ^1/2^, producing characteristic behaviors in the current (*J* ∝ *V*^3/2^), the potential versus position (φ ∝ *x*^4/3^), and the time-of-flight (τ_tof_ ∝ *V*^–3/2^). This deviates from the ohmic behavior where *v*_d_ ∝ φ; hence, *J* ∝ *V*, φ ∝ *x*, and τ_tof_ ∝ *V*^–1^, giving rise to the simpler form τ_tof_ = *L*^2^/*μV*. However, the classic ballistic regime applies only to vacuum,^31,32^ low-temperature,^33^ or short enough distance^34^ and time scale conditions.^35−38^ Hence, for more typical perovskite samples at room temperature, one can take the ballistic-like voltage-dependent mobility (BVM)^28^ regime that assumes instead *v*_d_ ∝ (dφ/d*x*)^1/2^, which results in *J* ∝ *V*^3/2^, φ ∝ *x*^5/3^, and τ_tof_ ∝ *V*^–3/2^. Considering the BVM formalism, the slow kinetics from our experiments would imply an ionic time-of-flight τ_tof,i_ ∝ *V*^–3/2^ due to a maximum ionic drift velocity *v*_d,i_ ∝ *V*^1/2^, allowing us to describe consistently the observed ionic time-of-flight values as^28^
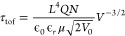
1where *L* is the distance between electrodes, ϵ_0_ the vacuum permittivity, ϵ_r_ the dielectric constant, *V*_0_ the onset potential for the BVM-regime, *Q* the charge, and *N* an effective homogeneous density of charge carriers (mostly mobile ions in this case). The τ_tof,i_ values calculated from eq 1 are shown in Figure 3 for a MAPbBr_3_ single crystal in conditions similar to those of the experiment. It can be seen that mobile ions with μ_i_ ≈ 10^–6^ cm^–2^V^–1^s^–1^ (from the IDD model) reproduce τ_tof,i_ ≈ 10^3^ s (from the experiment) with reasonably low values of charge concentration *N* ≈ 10^11^ cm^–3^ for a single-crystal sample. Here we have assumed unity for the charge *Q* as it corresponds to fast, bromide-related mobile ionic species. It is also noticeable that the electronic density calculated from the conductivity σ_e_ = 20 × 10^–8^ Ω^–1^ cm^–1^ results in values as low as *n* = 7 × 10^10^ cm^–3^, comparable with the ionic concentration estimated from Figure 3, i.e., *n* ≈ *N*, consistent with the IDD model outlined in section S2 in the Supporting Information. However, it should be noted that only ionic species acting as electronic dopants contribute to the increment in electronic conductivity, in such a way that *N* might just constitute a part of the total mobile ions. In contrast, electronic charge carriers with μ_e_ = 17 cm^2^ V^–1^ s^–1^, would require unrealistically large doping density *n* > 10^18^ cm^–3^ for a single crystal to relax in the order of ks. The different ways in which the drift velocity of electrons and ions relate to the voltage can explain the electronic currents following an ionic relaxation.

The electronic *J*_0_ values show nearly ohmic character, which may indicate that the electronic drift velocity is linear with the field. Then, one can take *v*_d,e_ ∝ *V* with kinetics in the range of μs, and a final bulk distribution of the electrostatic potential that is linear (φ ∝ *x*). The behavior of *v*_d,e_ is evidenced through electronic time-of-flight techniques in Figure S10, and a possible description of the potential is presented in the energy diagram of Figure S11. The ionic-induced band bending of the bulky perovskite as φ ∝ *x*^5/3^ could favor the charge collection during the relaxation period. At steady state, a seemingly ohmic behavior occurs in the bulk and a larger current is reported, possibly due to the IDD effect where the bias-induced accumulation of ions toward the interfaces eases the transport.

Our findings indicate that a coupling between ionic and electronic currents exists in such a way that the slower species (ions) condition the value of the measured current, which is actually determined by the faster carriers (electrons/holes). Consequently, ionic movement establishes the kinetics of the electronic response. Whatever the interplay mechanism is behind the current transients, it is clear from Figure 1a that homogeneous distribution of ions at zero bias hinders the charge extraction at short times, while the applied bias favors the current flowing at longer times. The latter effect can be possibly due to (i) an increment of the effective doping in the bulk perovskite via field ionization, (ii) a local increment of effective doping caused by ion redistribution (dynamic doping), (iii) a reduction of contact barriers toward the electrodes via electrode polarization, or (iv) a combination of these effects. Elucidation of the effective mechanism occurring in perovskite-based devices needs an exhaustive analysis of different structures (electrodes, active material, buffer layers, etc.) aimed at creating a coherent picture.

In summary, symmetrically contacted millimeters-thick MAPbBr_3_ samples were studied using a measurement protocol focused on the long time response toward steady-state current in cycles of biasing routines. This protocol has proven to be robust and reliable by delivering reproducible responses, regardless of possible ionic-related hysteresis and instability issues. The current transient upon bias application exhibits exponential increment. The behavior of the steady-state electronic drift currents suggests a seemly ohmic conductivity for electronic charge carriers. However, the most-likely ionic-related relaxation times follow a *t*_t_ ∝ *V*^–3/2^ trend up to the order of hours, which can be modeled with the formalism of the BVM regime of SCLC. Furthermore, we also studied the diffusion relaxation via IS measurements, which allowed us to estimate the ionic mobility by using the IDD model. By combining the BVM and the IDD models, it is suggested that the ionic currents are negligible in comparison to the total currents in our samples, for the measured bias and time ranges. The mobile ions, however, influence significantly the long-time electronic transport process via the modification of the charge density profile.

## Supporting Information Available

Sample preparation and structural, optical, and electrical data; outline of the ionic dynamic model and ballistic-like voltage-dependent mobility model (PDF)

## Notes

Data available on request from the authors.
