# Supplementary material for: Coupling between Ion Drift and Kinetics of Electronic Current Transients in MAPbBr3 Single Crystals
Source: ACS Energy Lett. 2022 Feb 11;7(3):946–51. doi: 10.1021/acsenergylett.1c02578 (PMC8922277; doi:10.1021/acsenergylett.1c02578)
Supplement: Supplementary file 1 — nz1c02578_si_001.pdf [file nz1c02578_si_001.pdf]

Supporting Information:

Coupling between Ion Drift and Kinetics of Electronic Current Transients in $MAPbBr_3$ Single Crystals

Marisé García-Batlle[1], Javier Mayén Guillén[2], Marian Chapran[3], Oriane Baussens[3], Julien Zaccaro[4], Jean-Marie Verilhac[2], Eric Gros-Daillon[3], Antonio Guerrero[1], Osbel Almora[1]* and Germà Garcia-Belmonte[1]*

[1] *Institute of Advanced Materials (INAM), Universitat Jaume I, 12006 Castelló, Spain*

[2] *Grenoble Alpes University, CEA, LITEN, DTNM, F38000 Grenoble, France*

[3] *Grenoble Alpes University, CEA, LETI, DOPT, F38000 Grenoble, France*

[4] *Grenoble Alpes University, CNRS, Grenoble INP, Institut Néel, F38042 Grenoble, France*

**Email:* almora@uji.es, garciag@uji.es

6 January 2022

Table S1 | Report on parameterization of ion migration in Hybrid Perosvkite Materials (~300 K)

**Methods**	**Architecture/thickness**	**Contacts/Composition**	**Parameters** μ_i: ion mobility ($cm^{-2}\,V^{-1}\,s^{-1}$) D_i: diffusion coeficient ($cm^{-2}\,s^{-1}$)	**Ref**
ToF spectroscopy and Monte Carlo simulation	SCs_2 mm-thick	Cr / **$MAPbBr_3$**/Cr<br>Au/ **$MAPbBr_3$**/Au	$\mu_i = 10^{-7}$	**[1]**
PL quenching method	film_350 nm	Au/**$MAPbI_3$**/Au	$\mu_i = 9.1 \times 10^{-7}$<br>$D_i$= 5 to 6 x 10^{-9}	**[2]**
Nuclear magnetic resonance (NMR) spectroscopy	PCs_1 mm-thick	Gr/**$MAPbI_3$**/Gr	$D_i = 2 \times 10^{-9}$	**[3]**
Step-dwell-step-probe (SDSP) photocurrent transient.	film_400 nm	ITO/PTAA/**FAPI**/C60/BCP/Ag	$\mu_i = 3 \times 10^{-7}$	**[4]**
Photothermal induced resonance (PTIR) microscopy	film_300 nm	Au/**$MAPbI_3$**/Au	$\mu_i = 1.5 \times 10^{-9}$	**[5]**
Chronoamperometry measurements	PCs_1mm-thick	Pt/**$MAPbI_3$**/Cr	$D_i = 3 \times 10^{-8}$<br>$\mu_i = 10^{-6}$	**[6]**

Impedance spectroscopy (IS)	PCs_0.6 mm-thick	Gr/**$MAPbI_3$**/Gr	D_i= 2.4 x 10^{-8}	**[7]**
	SCs_1μm-thick	TiO2/**$MAPbBr_3$**/Au	D_i= 1.8 x 10^{-8}	**[8]**
	SCs_1mm-thick	Cr / **$MAPbBr_3$**/Cr	D_i= 2.6 x10^{-8}<br>μ_i= 1 x 10^{-6}	**[9]**
Chronoamperometry measurements and Impedance spectrosocopy*	SCs_1-2 mm	Cr / **$MAPbBr_3$**/Cr	D_i= 3.1 x 10^{-8}<br>μ_i= 1.2 x 10^{-6}	**this work**

Column 2 lists the morphology of the perovkite devices being SCs: Single Crystals, PCs: Polycrystals

S1. Further experiments

METHODS

I. $MAPbBr_3$ SCs growth & device fabrication

$MAPbBr_3$ SCs were grown via Modified Inverse Temperature Crystallization (MITC) method. The growth process was similar to the one described in a previous work [10] using linear 5°C/h temperature ramp. A seed was first obtained by spontaneous nucleation, and used subsequently for crystal growth. At the end of the growth process, SCs of 4 mm × 4 mm × 2 mm on average were obtained. They were mechanically polished to mirror grade quality and Cr electrodes were thermally evaporated on opposite sides.

II. XRD

A D8 Endeavor diffractometer equipped with a Johanssonn monochromator working in Bragg-Brentano θ-2θ geometry was used (Cu K_α λ = 1.5406 Å). An acquisition time of 2.5 s was set using 0.01° step, 2θ values ranging from 5° to 90°.

III. UV-Visible spectroscopy & Tauc plot

For transmittance data, a Perkin Elmer Lambda 900 UV-Vis-NIR spectrometer was used with a wavelength range of 400-600 nm, a 0.1 nm step and equipped with a Tungsten lamp. Tauc plot were charted using equation 1, 2, 3 and 4, where α is the absorption coefficient, h is the Planck constant, ν is the photon frequency, E_g is the band gap energy, B is a constant, γ is a factor depending on the nature of the electron transition and equal to 1/2 or 2 for the direct and indirect transition band gaps (here γ =1/2),[11] c is the speed of light, A is the absorbance, T is the transmittance, d is the sample thickness and e is the elementary charge. In the Tauc plot, E_g was extracted at the intersection between the x axis and the linear fitting of the linear part in the onset region.

$$(\alpha h\nu)^{\frac{1}{\gamma}} = B(h\nu - E_g) \tag{S1}$$

$$h\nu = \frac{hc}{\lambda 10^{-9} e} \tag{S2}$$

$$\alpha = \frac{\ln(10)A}{d} \tag{S3}$$

$$A = -\log T \tag{S4}$$

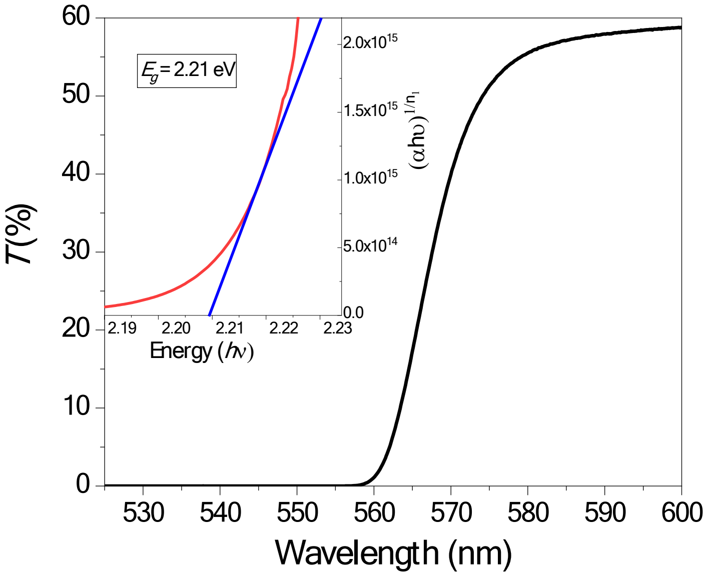

Figure S1. Transmittance spectre of $MAPbBr_3$ SC via UV-visible spectroscopy (SC thickness = 0.97 mm) In the inset : Tauc plot of $MAPbBr_3$ SC for band gap determination (E_g = 2.21 eV)

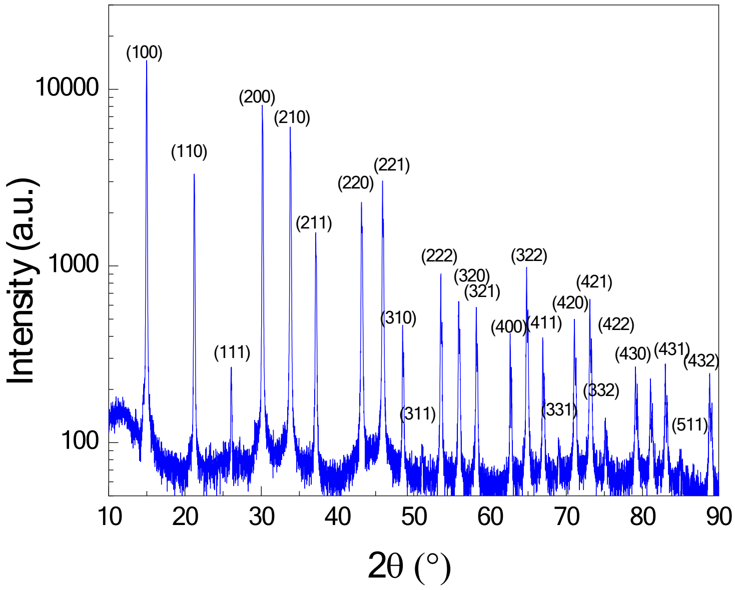

Figure S2. PXRD diffractogram in log scale of crushed $MAPbBr_3$ SC showing cubic crystal lattice (lattice parameter = 5.928 ± 0.003 Å) and no other parasitic phases.

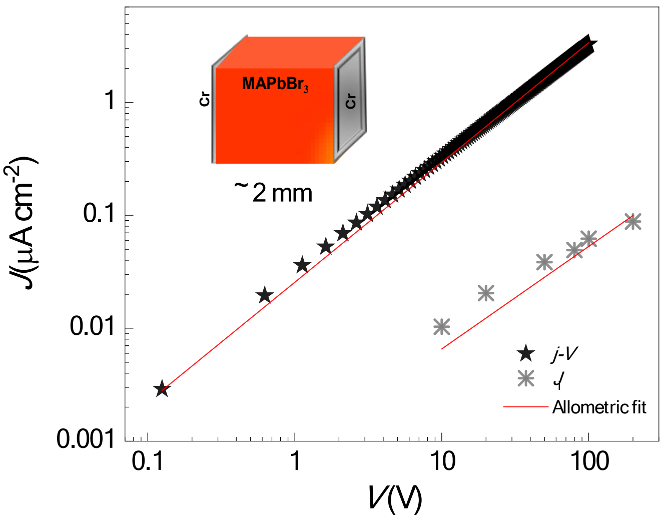

Figure S3. Comparation between the current–voltage characteristics (*j*-*V*) with scan rate of 90 mV/s and step: 1 V and the ionic drift currents J_i immediately obtained after bias removal of 2 mm-thick $MAPbBr_3$ SC symmetrically contacted with Cr electrodes It is remarkable the ohmic character of the characteristics *j*-*V* curve, in agreement with previous analysis on Cr-contacted perovskite device.[9, 12, 13]

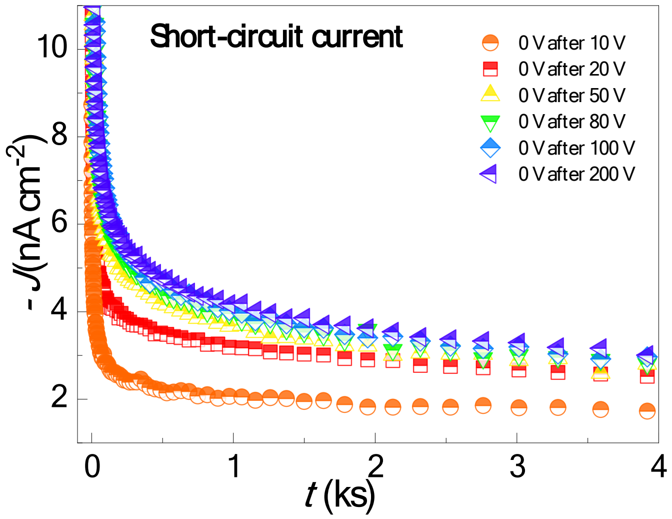

Figure S4. a) Current transient response to short-circuit condition (0 V-bias voltage) of a $MAPbBr_3$ SC symmetrically contacted with evaporated Cr electrodes.

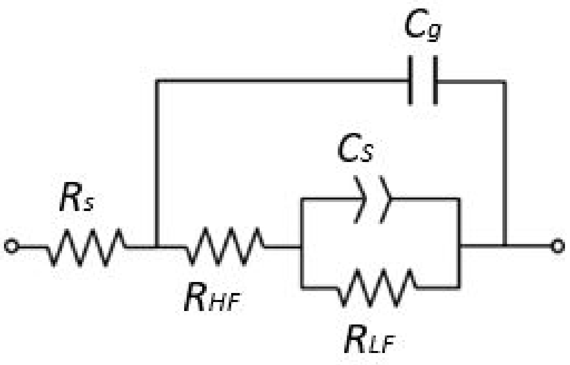

Figure S5. Equivalent circuit used to fits the impedance spectra: with series resistance R_s, C_g geometric capacitance and R_{HF}, and R_{LF} the high-, and low-frequency resistances, respectively. The low-frequency capacitance usually labelled as surface capacitance C_s.

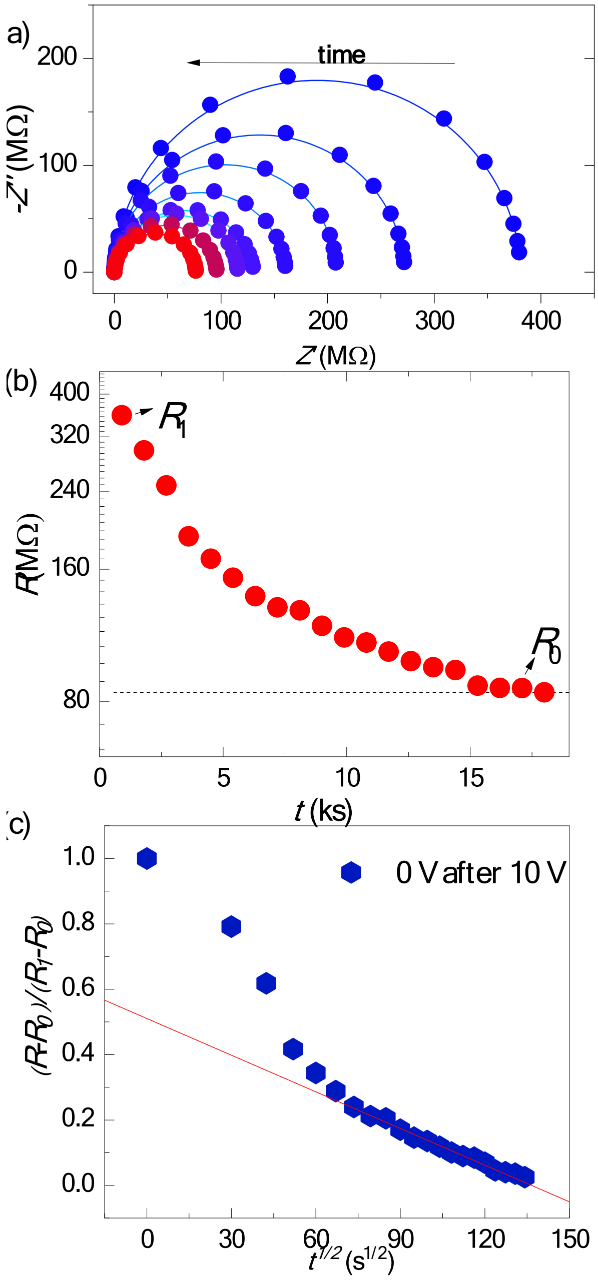

Figure S6. Variation of the impedance after removing 10 V bias and evolution with time b)Variation of the resistance extracted from fitting impedance spectra after bias removal at t = 0. Note the log scale in the vertical axis that informs on the complex function (non-exponential) of the resistance variation c) Variation of resistance ratio as a function of time following the relationship in equation (5) Solid line represents a linear fitting.

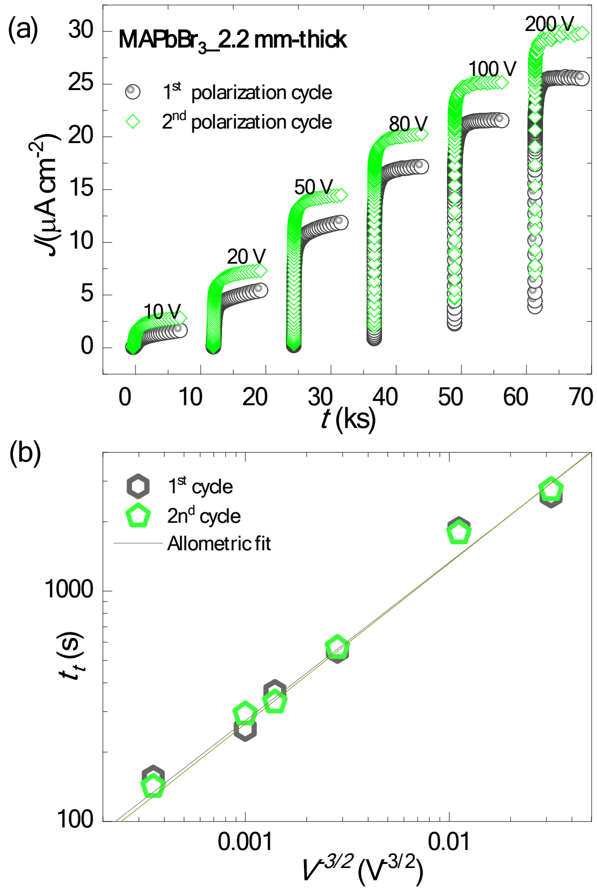

Figure S7. a) Long-time current transient's response to voltage steps of a 2.2 mm thick $MAPbBr_3$ SC- symmetrically contacted with evaporated Cr eelctrodes and b) corresponding characteristic ionic relaxation time constants as a function of the applied voltage.

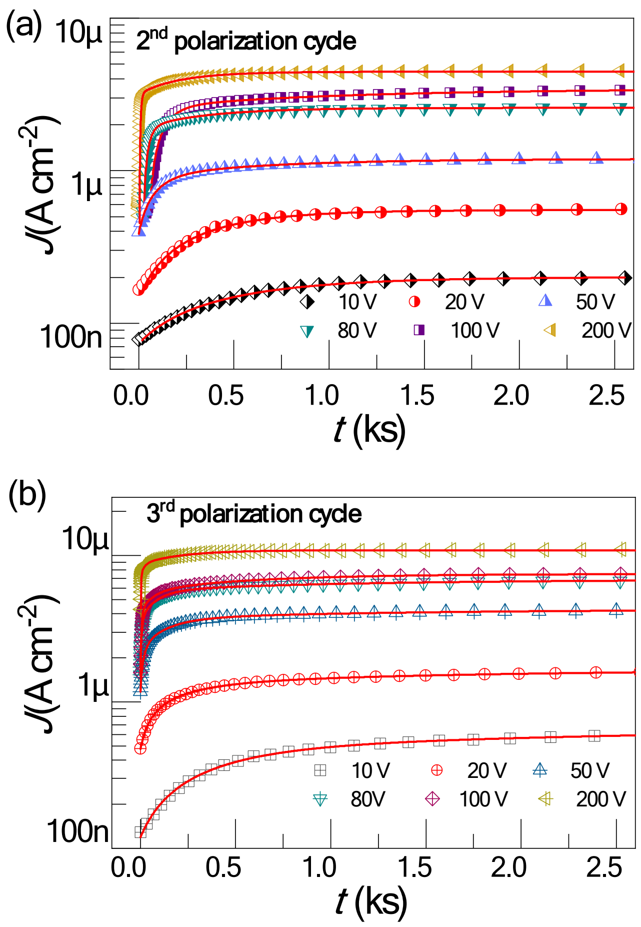

Figure S8. a) Long-time current transient response to different voltage steps during the 2nd and b) 3rd cycle of measurement of ~2 mm-thick $MAPbBr_3$ SC.

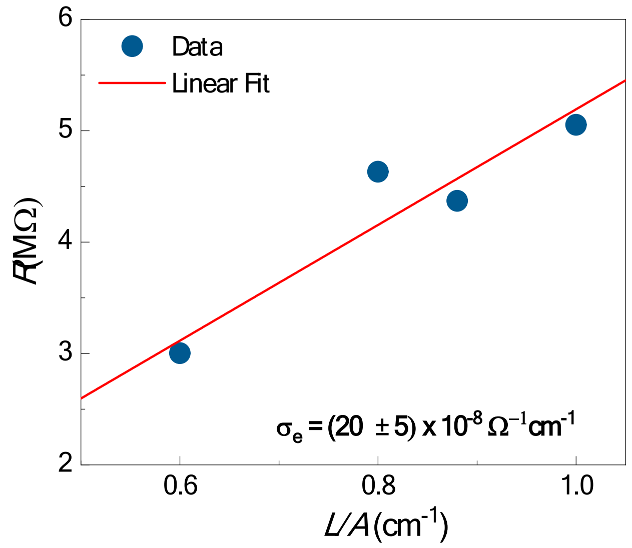

Figure S9. Scaling of the high-frequency resistance for four samples of different thicknesses and electrode area ratio, exhibiting linear relationship with geometrical parameter

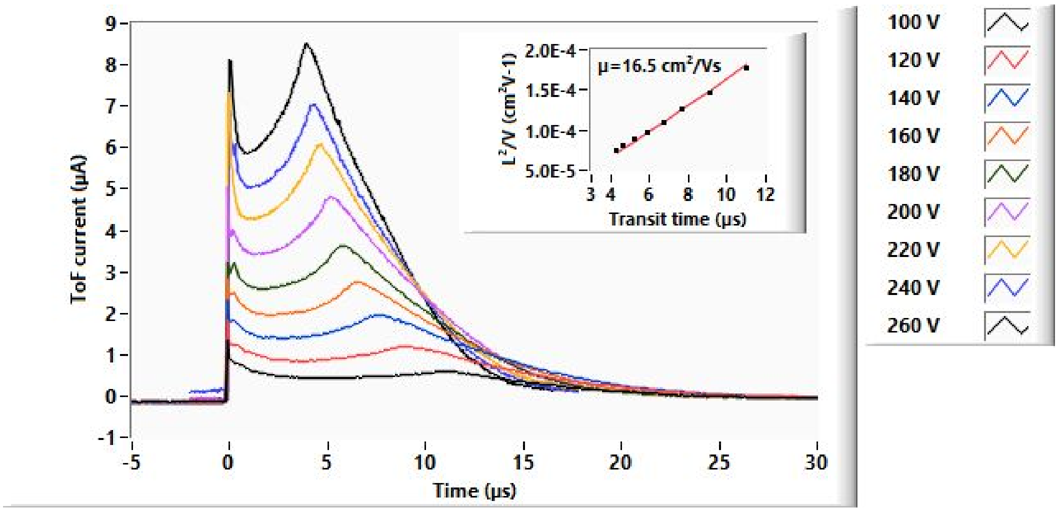

Figure S10. Hole time-of-flight (ToF) for a $MAPbBr_3$ single crystal (thickness 1.33 mm)

S2. Ionic Dynamic Doping (IDD) model

The IDD model,[6, 9] relies on the way the redistribution of ionic species along the layer bulk modulate the electronic carrier density. Mobile ions acts as dopants and locally alter the doping density by a dynamic doping process. Therefore, the resistance variation can be explained in terms of the increase in electronic carrier density. A detailed view of the high-frequency resistance decrese under 0-V bias can be observed in Figure S6a. By extracting the fitting parameters in Figure S6b the resistance shows a large initial value R_1 at shorter relaxing times and a steady-state value R_0 (background state) after a long equilibration time. A simple expression based in the IDD model[6] is thereby derived where R_1 and R_0 are the limiting resistances which can be normalized by the resistance step $(R_1 - R_0)$

$$\frac{R - R_0}{R_1 - R_0} = 1 - \frac{s}{L} \tag{S5}$$

where L and s stands for the sample thickness and the width of the high doping region respectively. The normalized resistance is formulated as a linear function of s giving rise to the expression (S5), that again predicts a linear dependence of the sample resistance with the width of the high doping zone s.

Let us also assume that ions initially accumulate following a narrow distribution near the contact with w standing for the distribution width. The accumulation occurs by effect of the applied electrical field. This ionic accumulation is expected by formation of a diffusion ion layer in the vicinity of the contacts and by very limited interfacial chemical interaction within the experimental time framework. Mobile ions depleted from the layer bulk induce a sort of electronic de-doping, reducing as a consequence the doping level and producing higher resistance. When bias is removed, ions are released and tend to diffuse back to their equilibrium position in such a way that the mean square displacement of the ion distribution enlarges with time. The simple outlined model would entail diffusion takes place in 1D ($\delta = 1$), but the real situation might be more complex with diffusion dimensionality approaching larger values because of the polycrystalline structure. For the sake of simplicity, let's assume that the distribution width spreads by diffusion as

$$w = \sqrt{2\delta D t} \tag{S6}$$

with D accounts for the ion diffusion coefficient, $\delta = 1$ for 1D diffusion, and t is the time. Equation S6 informs on how the high doping region width s extends within the perovskite layer bulk as the ion distribution spreads to attain initial homogeneity.

An additional component of the model concerns the relation between the width of the high doping zone s and the spead of the ion distribution w. For a Gaussian distribution, one can observe that nearly 99% of the ions concentrates within $3w$ as to assume that $s = 3w$. We recognize that this is in fact an oversimplification of a complex problem that should include concrete ion distributions and how they influence the local doping density. In any case, one can derive a simple linear expresion for the variation of the resistance that suffices for our purposes.

By combining Equation (S5) and (S6), and $\delta = 1$ an expression for the time dependence of the normalized resistance step results as

$$\frac{R - R_0}{R_1 - R_0} = 1 - \frac{3\sqrt{2Dt}}{L} \tag{S7}$$

which predicts a linear dependence as $\propto \sqrt{t}$ with slope determined by the diffusion coefficient. One can also obtain a value for the ion diffusion coefficient by assuming the Einstein's relation, equation

$$\mu_{ion} = \frac{q\, D_{ion}}{k_B T} \tag{S8}$$

where q is the elementary charge, k_B stands for the Boltzmann constant, and T is the absolute temperature. It's important to mentioned that due to the simplicity related to the model, a deviation occurs at shorter times, presumably unable to capture second order depolarization effects for greater moving ion concentrations.

S3. Ballistic-like voltage-dependent mobility (BVM) model of space-charge-limited current (SCLC)

In the classic mobility regime of SCLC, the drift velocity is a function of the electric field ξ and the bias- and space-independent constant mobility μ as

$$v_d = \mu_0 \xi \tag{S9}$$

Equation (S9) results in the Mott-Gurney law[14] where the current density is quadratic with the external applied voltage ($J \propto V^2$), the electrostatic potential behaves with the position as $\varphi \propto x^{3/2}$ and the time of flight can be approximated to $\tau_{tof} \propto V^{-2}$.

In the BVM model,[15] the mobility is taken as function of the field, and thus the bias, as

$$\mu_0 = \mu \sqrt{\frac{V_0}{L\xi}}. \tag{S10}$$

where L is the distance between electrodes, V_0 is the onset voltage for the BVM regime, μ is the threshold mobility for the transition between ohmic and SCLC regime and the absolute field value is considered as $|\xi| = |d\varphi/dx|$ at each position x in between the elecctrodes and $|\xi| \approx V/L$ at the active electrode. A deduction with an expression for V_0 is presented in the original publication[15] under two main assumptions: (i) the larger L_i the larger μ, where L_i is Frenkel's equation[16] for the distance between the ions and their local potential maxima upon application of an external field; and (ii) the smaller L_D the larger μ, where L_D is a Debye length for the accumulation of mobile ions towards the electrodes.

By subsituting Equation (S10) in (S9), the BVM drift velocity results as

$$v_d = \mu \sqrt{\frac{V_0}{L}\xi} \tag{S11}$$

Equation (S10) can also be approximated as a particular case of Poole-Frenkel[16-18] ionized-trap-mediated transport when field dependent charge carrier density $n \propto \xi$ and for narrow ranges of ξ.[15] Furthermore, Equation (S10) can be substituted in the expression for the associated total current density

$$J = Q\, n\, v_d \tag{S12}$$

where Q charge. Taking N from equations (S12) and (S10), the Poisson equation can be solved for $v_d \propto (d\varphi/dx)^{1/2}$ trend such as that of Equation (S10), which results in a potential

$$\varphi = \frac{3}{5}\left(\frac{3}{2}\right)^{\frac{2}{3}}\left(\frac{J}{\epsilon_0\epsilon_r\mu}\sqrt{\frac{L}{V_0}}\right)^{\frac{2}{3}} x^{\frac{5}{3}} \tag{S13}$$

The possible effect of a potential as Equation (S13) is presented in Figure S11. Moreover, after evaluating Equation (S13) at $x = L$ where $\varphi = V$, and since $\sqrt{500/243} \approx \sqrt{2}$, the current density results

$$J = \frac{\epsilon_0 \epsilon_r \mu}{L^3} \sqrt{2V_0}\, V^{3/2} \tag{S14}$$

where ϵ_0 is the vacuum permittivity and ϵ_r the dielectric constant. Subsequently, from the definition of time of flight

$$\tau_{tof} = \frac{L}{v_d} \tag{S15}$$

one can substitute (S14) and (S12) in (S15) to obtain the BVM approximation of the time of flight as

$$\tau_{tof} = \frac{9L^4 QN}{4\epsilon_0 \epsilon_r \mu \sqrt{V_0}} V^{-3/2} \tag{S16}$$

where N is now an effective homogeneous charge carrier density. Typical τ_{tof} values of the BVM model are presented in Figure 3a of the main manuscript.

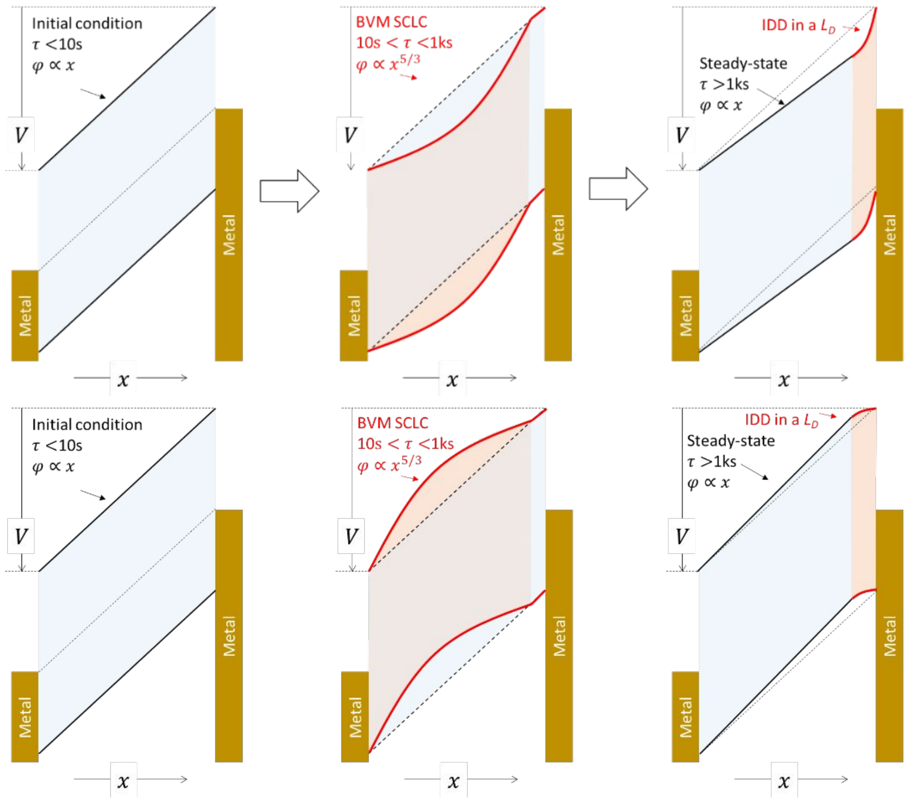

Figure S11. Proposed energy diagram of the evolution of transport for electron (top) and hole (bottom) charge carriers. From an initially ohmic behaviour, the mobile ions modify the space charge to create BVM SCLC within characteristic times ~ks and subsequently pile up towards the electrodes resulting in a bulkily ohmic transport eased by interface ionic dynamic doping.

References

[1] Artem Musiienko, Jindřich Pipek, Petr Praus, Mykola Brynza, Eduard Belas, Bogdan Dryzhakov, Mao-Hua Du, Mahshid Ahmadi, Roman Grill, Deciphering the effect of traps on electronic charge transport properties of methylammonium lead tribromide perovskite, *Sci. Adv.*, 6, eabb6393, https://doi.org/10.1126/sciadv.abb6393

[2] C. Li, A. Guerrero, S. Huettner, J. Bisquert, Unravelling the Role of Vacancies in Lead Halide Perovskite through Electrical Switching of Photoluminescence, *Nat. Commun.* **2018**, 9, 5113,

[3] A. Senocrate, I. Moudrakovski, G. Y. Kim, T. Y. Yang, G. Gregori, M. Grätzel, J. Maier, The Nature of Ion Conduction in Methylammonium Lead Iodide: A Multimethod Approach, *Angew. Chem., Int. Ed.* **2017**, 56, 7755,

[4] L. Bertoluzzi, R. A. Belisle, K. A. Bush, R. Cheacharoen, M. D. McGehee, B. C. O'Regan, In Situ Measurement of Electric-Field Screening in Hysteresis-Free PTAA/FA0.83Cs0.17Pb(I0.83Br0.17)3/C60 Perovskite Solar Cells Gives an Ion Mobility of ~3 × 10-7 cm2/(V s), 2 Orders of Magnitude Faster than Reported for Metal-Oxide-Contacted Perovskite Cells with Hysteresis, *J. Am. Chem. Soc.* **2018**, 140, 12775, https://doi.org/10.1021/jacs.8b04405

[5] Yongbo Yuan, Jungseok Chae, Yuchuan Shao, Qi Wang, Zhengguo Xiao, Andrea Centrone, Jinsong Huang, Photovoltaic Switching Mechanism in Lateral Structure Hybrid Perovskite Solar Cells, *Adv. Energy Mater.* **2015**, 5, 1500615, https://doi.org/10.1002/aenm.201500615

[6] Marisé García-Batlle, Sarah Deumel, Judith E. Huerdler, Sandro F. Tedde, Antonio Guerrero, Osbel Almora, Germà Garcia-Belmonte, Mobile Ion-Driven Modulation of Electronic Conductivity Explains Long-Timescale Electrical Response in Lead Iodide Perovskite Thick Pellets, *ACS Appl. Mater. Interfaces* **2021**, 13, 35617, https://doi.org/10.1021/acsami.1c06046

[7] Tae-Youl Yang, Giuliano Gregori, Norman Pellet, Michael Grätzel, Joachim Maier, The Significance of Ion Conduction in a Hybrid Organic–Inorganic Lead-Iodide-Based Perovskite Photosensitizer, *Angew. Chem., Int. Ed.* **2015**, 54, 7905, https://onlinelibrary.wiley.com/doi/abs/10.1002/anie.201500014

[8] Wei Peng, Clara Aranda, Osman M. Bakr, Germà Garcia-Belmonte, Juan Bisquert, Antonio Guerrero, Quantification of Ionic Diffusion in Lead Halide Perovskite Single Crystals, *ACS Energy Lett.* **2018**, 3, 1477, https://doi.org/10.1021/acsenergylett.8b00641

[9] Marisé García-Batlle, Oriane Baussens, Smaïl Amari, Julien Zaccaro, Eric Gros-Daillon, Jean-Marie Verilhac, Antonio Guerrero, Germà Garcia-Belmonte, Moving Ions Vary Electronic Conductivity in Lead Bromide Perovskite Single Crystals through Dynamic Doping, *Adv. Electron. Mater.* **2020**, 6, 2000485, https://doi.org/10.1002/aelm.202000485

[10] Smaïl Amari, Jean-Marie Verilhac, Eric Gros D'Aillon, Alain Ibanez, Julien Zaccaro, Optimization of the Growth Conditions for High Quality CH3NH3PbBr3 Hybrid Perovskite Single Crystals, *Crystal Growth & Design* **2020**, 20, 1665, https://doi.org/10.1021/acs.cgd.9b01429

[11] Patrycja Makuła, Michał Pacia, Wojciech Macyk, How To Correctly Determine the Band Gap Energy of Modified Semiconductor Photocatalysts Based on UV–Vis Spectra, *J. Phys. Chem. Lett.* **2018**, 9, 6814, https://doi.org/10.1021/acs.jpclett.8b02892

[12] Oriane Baussens, Loli Maturana, Smaïl Amari, Julien Zaccaro, Jean-Marie Verilhac, Lionel Hirsch, Eric Gros-Daillon, An insight into the charge carriers transport properties and electric field distribution of CH3NH3PbBr3 thick single crystals, *Appl. Phys. Lett.* **2020**, 117, 041904, https://doi.org/10.1063/5.0011713

[13] Jeremy T. Tisdale, Eric Muckley, Mahshid Ahmadi, Travis Smith, Cody Seal, Eric Lukosi, Ilia N. Ivanov, Bin Hu, Dynamic Impact of Electrode Materials on Interface of Single-Crystalline Methylammonium Lead Bromide Perovskite, *Adv. Mater. Interfaces* **2018**, 5, 1800476, https://doi.org/10.1002/admi.201800476

[14] N.F. Mott, R.W. Gurney, *Electronic Processes in Ionic Crystals*, Clarendon Press, California, USA **1940**.

[15] Osbel Almora, Daniel Miravet, Marisé García-Batlle, Germà Garcia-Belmonte, Ballistic-like Space-charge-limited Currents in Halide Perovskites at Room Temperature, *Appl. Phys. Lett.* **2021**, 119, 242107, https://aip.scitation.org/doi/abs/10.1063/5.0076239

[16] J. Frenkel, On Pre-Breakdown Phenomena in Insulators and Electronic Semi-Conductors, *Phys. Rev.* **1938**, 54, 647, https://doi.org/10.1103/PhysRev.54.647

[17] Simon M. Sze, Kwok K. Ng, *Physics of Semiconductor Devices*, John Wiley & Sons, Hoboken, New Jersey, USA **2007**.

[18] P. N. Murgatroyd, Theory of space-charge-limited current enhanced by Frenkel effect, *J. Phys. D: Appl. Phys.* **1970**, 3, 151, https://doi.org/10.1088/0022-3727/3/2/308
